# Aging Gut Microbiota at the Cross-Road between Nutrition, Physical Frailty, and Sarcopenia: Is There a Gut–Muscle Axis?

**DOI:** 10.3390/nu9121303

**Published:** 2017-11-30

**Authors:** Andrea Ticinesi, Fulvio Lauretani, Christian Milani, Antonio Nouvenne, Claudio Tana, Daniele Del Rio, Marcello Maggio, Marco Ventura, Tiziana Meschi

**Affiliations:** 1 Microbiome Research Hub, University of Parma, Parco Area delle Scienze 11/A, 43124 Parma, Italy; andrea.ticinesi@unipr.it (A.T.); christian.milani@unipr.it (C.M.); anouvenne@ao.pr.it (A.N.); daniele.delrio@unipr.it (D.D.R.); marco.ventura@unipr.it (M.V.); tiziana.meschi@unipr.it (T.M.); 2 Department of Medicine and Surgery, University of Parma, Via Antonio Gramsci 14, 43126 Parma, Italy; ctana@ao.pr.it (C.T.); marcellomaggio2001@yahoo.it (M.M.); 3 Dipartimento Medico-Geriatrico-Riabilitativo, Azienda Ospedaliero-Universitaria di Parma, Via Antonio Gramsci 14, 43126 Parma, Italy; 4 Laboratory of Probiogenomics, Department of Chemistry, Life Sciences and Environmental Sustainability, University of Parma, Parco Area delle Scienze 11/A, 43124 Parma, Italy; 5 Laboratory of Phytochemicals in Physiology, Department of Food and Drug Science, University of Parma, Parco Area delle Scienze 27/A, 43124 Parma, Italy

**Keywords:** diet, physical frailty, short-chain fatty acids, metagenomics, geriatrics

## Abstract

Inadequate nutrition and physical inactivity are the mainstays of primary sarcopenia–physiopathology in older individuals. Gut microbiota composition is strongly dependent on both of these elements, and conversely, can also influence the host physiology by modulating systemic inflammation, anabolism, insulin sensitivity, and energy production. The bacterial metabolism of nutrients theoretically influences skeletal muscle cell functionality through producing mediators that drive all of these systemic effects. In this study, we review the scientific literature supporting the concept of the involvement of gut microbiota in primary sarcopenia physiopathology. First, we examine studies associating fecal microbiota alterations with physical frailty, i.e., the loss of muscle performance and normal muscle mass. Then, we consider studies exploring the effects of exercise on gut microbiota composition. Finally, we examine studies demonstrating the possible effects of mediators produced by gut microbiota on skeletal muscle, and intervention studies considering the effects of prebiotic or probiotic administration on muscle function. Even if there is no evidence of a distinct gut microbiota composition in older sarcopenic patients, we conclude that the literature supports the possible presence of a “gut–muscle axis”, whereby gut microbiota may act as the mediator of the effects of nutrition on muscle cells.

## 1. Introduction: Sarcopenia and Physical Frailty in Aging

### 1.1. The Clinical Context

Sarcopenia is a geriatric syndrome with a high prevalence in older individuals; its presence is estimated in up to 35% of hospital wards [1,2]. The primary form of sarcopenia is an age-related condition with multiple and often unknown causes, which is exacerbated by acute and chronic disease, inflammation, endocrine dysfunction, physical inactivity, and unhealthy lifestyle [3]. It must be distinguished from secondary sarcopenia, which is etiologically associated with some chronic diseases leading to persistent inflammation or mobility limitations, namely Chronic Obstructive Pulmonary Disease (COPD), heart failure, rheumatoid arthritis, connectivitis, and cirrhosis [4,5].

According to the European Working Group on Sarcopenia in Older People (EWGSOP), the cornerstones for sarcopenia definition are muscle mass depletion and the reduction of muscle performance, both resulting from boosted protein catabolism or anabolic resistance [6]. As such, to meet EWGSOP diagnostic criteria, sarcopenic patients must have a reduced appendicular lean mass, which is measured by dual-energy X-ray absorptiometry (DXA), or a reduced skeletal muscle index, and derived through bioimpedance analysis (BIA) and altered physical tests. These tests estimate muscle function and include handgrip strength measured by a hand-held dynamometer, and Short Physical Performance Battery (SPPB) [6].

Sarcopenia may significantly overlap with physical frailty, although the two conditions are distinct [7]. Frailty has been defined as a condition of increased vulnerability after a stressor event, increasing the risk of adverse outcomes [8]. This concept has been operationalized according to two distinct models, i.e., phenotypic and deficit accumulation [8]. Both models stress the importance of a reduction of physical performance and muscle strength in the physiopathology of frailty, and this aspect is the main feature of frail persons with sarcopenia. The most used clinical tools for diagnosing frailty are the Fried criteria (phenotypic model) and the Rockwood frailty index or Clinical Frailty Scale (deficit accumulation model) [8]. Recently, the concept of physical frailty has been also operationalized as the condition of patients experiencing reduced physical performance (i.e., SPPB score between 3/12 and 9/12) [9]. As such, patients with physical frailty may be also diagnosed with sarcopenia if their appendicular lean mass, measured by DXA, or skeletal muscle index, measured by BIA, fall within the range established by EWGSOP [9].

Although the precise causes of primary sarcopenia are not yet identifiable, significant alterations of endocrine–metabolic parameters, such as low insulin-like growth factor-1 (IGF-1) or free testosterone levels, often occur in sarcopenic patients [10]. Chronic subclinical inflammation and insulin resistance also have pivotal roles in promoting anabolic resistance in myocytes [11]. The main consequence is reduced muscle protein synthesis, which also depends on ultrastructural modifications in the number and functionality of mitochondria and intracellular lipid deposition [12,13]. Anabolic resistance also promotes adipose infiltration in the muscle, which contributes to decreased muscle functionality [14,15].

From a clinical perspective, sarcopenia is associated with detrimental clinical consequences, particularly in the oldest old. A recent meta-analysis by Beaudart et al. [16] has shown that sarcopenic individuals have a significantly increased risk of death, mobility disability, falls, and hospital admissions in a follow-up period ranging from three months to 9.8 years. Sarcopenia is also associated with a poor quality of life independently of adverse health outcomes, particularly in those patients with very low muscle strength [17]. Interestingly, sarcopenia can represent both a risk factor for hospitalization, since it is highly prevalent in older patients admitted to hospitals [2], and an adverse event of hospitalization itself, particularly when the stay is prolonged and characterized by forced bed rest [18].

### 1.2. Treatment and Prevention of Sarcopenia: Nutrition and Exercise

Elderly individuals generally experience a decline of nutrient and energy intake with increasing age [19,20]. This phenomenon is generally due to age-related loss of appetite, the so-called “anorexia of aging”, whose physiopathology is only partly understood [21]. It may also depend on increased energy requirements due to acute or chronic inflammation, leading to “disease-related malnutrition” [22].

Malnutrition and sarcopenia often overlap in older patients [23], so that one of the mainstays of sarcopenia prevention and treatment is promoting adequate nutrition [24]. The prescription of adequate intakes of proteins, vitamin D, antioxidant nutrients, and long-chain polyunsaturated fatty acids has been particularly emphasized in this field [25], since these nutrients are able to counteract anabolic resistance, promote protein synthesis, and modulate inflammation, thereby preventing its detrimental consequences on muscle cells [25]. The protein intake recommended for older individuals with malnutrition or sarcopenia is even higher (1.2–1.5 g/kg/day) than that of healthy-active subjects (1.0–1.2 g/kg/day), in order to meet the increased energy requirements and overcome the loss of lean mass [26].

The other mainstay of sarcopenia prevention and treatment is physical exercise [27]. In older individuals, strength training is able to promote the differentiation and proliferation of muscle satellite cells, the energy production and metabolic efficiency of mitochondria, muscle capillarity with improved oxygen delivery, innervation, and metabolic-sensing pathways, all of which lead to improved anabolism and insulin sensitivity [28]. As such, almost all clinical trials have demonstrated the positive effects of exercise in regard to preventing sarcopenia [29].

However, according to a recent systematic review, studies that have explored the combined effect of nutritional intervention with exercise have demonstrated only minor additional effects of nutrition [29]. This is probably due to the different study designs and settings (hospital vs. long-term care vs. community). The wide heterogeneity of nutritional regimens and supplements studied, and the lack of interventions targeted on nutritional requirements, are also relevant issues. In fact, from a modern perspective, the treatment of sarcopenia requires multidomain interventions, with personalized nutritional support targeted at individual patient needs [30].

### 1.3. Aims

The aim of the present paper was to review the current state-of-the-art literature supporting the hypothesis that gut microbiota is involved in the physiopathology of sarcopenia and physical frailty in aging, and also focusing on the role of diet.

## 2. Gut Microbiota: The Neglected Actor in Aging?

### 2.1. General Concepts about Gut Microbiota across the Lifespan

The human gut microbiota is composed of as much as 10^14^ bacteria, viruses, fungi, protozoa, and Archaea, with a gene pool 150 times larger than that of the host, and a weight esteemed between 175 g and 1.5 kg [31,32,33]. It establishes a symbiotic relationship with the host, whereby individual environmental and genetic factors can shape its composition, while the host physiology is influenced and gets adapted to its presence [34]. In healthy individuals, the gut microbiome generally includes between 1100 and 2000 bacterial taxa, most of which cannot be cultivated with traditional microbiological techniques [35]. The recent availability of culture-independent metagenomic approaches, which are based on the high-throughput sequencing of bacterial DNA extracted from feces, and the subsequent identification of 16S ribosomal-RNA (rRNA) gene polymorphisms (16S rRNA microbial profiling) [36], has boosted research on the human gut microbiota [37], and helped identify fecal microbiota alterations that are associated with a large number of diseases [38,39].

The gut microbiota composition is generally shaped in early childhood, depending on geographical factors, the type of delivery, breastfeeding, age of weaning, antibiotic exposure, and dietary regimens [40,41]. By the age of three years old, the gut microbiota reaches its mature composition, which is maintained relatively stable over the lifespan, even in front of perturbative events such as moderate lifestyle changes, acute diseases, and antibiotic treatments [42,43]. The healthy adult gut microbiota includes bacteria belonging to 10 phyla, even if two phyla—*Bacteroidetes* and *Firmicutes*—account for as much as 99% of species [42]. Generally, the average relative abundance of *Bacteroidetes* is inversely proportional to that of *Firmicutes*, and vice versa [42]. In fact, a pronounced interindividual variability is present. Arumugam et al. [44] even identified some typical clusters of fecal microbiome composition that are recurring in the healthy population. These clusters, called “enterotypes”, may partly depend on dietary habits. For example, the *Prevotella* enterotype, which is characterized by a high representation of *Prevotella* spp., has been associated with high-carbohydrate, high-fiber diets [44].

### 2.2. Diet as Determinant of Gut Microbiota Composition

At present, geographical location [45] and diet [46] are the major environmental factors explaining the interindividual differences in healthy gut microbiota composition. In a population-based Dutch cohort of 1135 adults, metagenomic analyses on fecal samples revealed significant correlations between as many as 60 dietary parameters and interindividual microbiome variability [47]. Acute shifts of dietary habits towards high-protein diets are associated with low microbial diversity; an increased representation of bacteria with tolerance to biliary acids, such as *Bacteroides*, *Alistipes*, and *Bilophila*; and decreased representation of bacteria able to metabolize vegetal polysaccharides, such as *Roseburia*, *Eubacterium*, and *Ruminococcus* [48]. Conversely, vegan diets are associated with an over-representation of *Prevotella* and high microbial diversity [48]. However, long-term consumption of high amounts of animal proteins has been associated with favorable gut microbiota compositions, especially when these habits are associated with physical exercise [49].

Meanwhile, high-fat diets have been associated with detrimental consequences on the gut microbiota. These diets generally promote decreased representation of *Bacteroidetes* and an overgrowth of *Firmicutes*, including a wide range of opportunistic pathogens. These changes enhance gut mucosa permeability and promote systemic inflammation, subclinical immune activation, and metabolic derangements towards insulin resistance [50]. In this context, the relative ratio of *Bacteroides* and *Prevotella* has even been proposed as a biomarker of healthy and active aging, diet, and lifestyle [51].

Several studies have also recently demonstrated that adherence to a Mediterranean-style diet is associated with beneficial gut microbiota characteristics, including higher biodiversity, over-representation of *Prevotella*, and under-representation of opportunistic pathogens [52,53,54,55].

Intervention studies have demonstrated that fecal microbiota composition is much more influenced by long-term dietary patterns, rather than by temporary changes in food intake, due to the high level of resilience of the bacterial community [56]. However, a change in the intake of specific nutrients, such as non-digestible starches, inulin, fructooligosaccharides, and polyunsaturated fatty acids, may be associated with the significant over-expression of some “minor players”, such as *Eubacterium rectale*, bifidobacteria, and *Oscillibacter* [57,58,59].

### 2.3. The Gut Microbiota in Aging

After the age of 65, gut microbiota resilience is generally reduced, so that its overall composition is more vulnerable to lifestyle changes, drug treatments such as antibiotics, and disease [60,61]. As a result, species richness (i.e., the number of taxa that metagenomic analyses are able to identify in fecal samples) is reduced, and interindividual variability is enhanced [60,61]. In an Irish population-based study, Claesson et al. showed that gut microbiota biodiversity is inversely correlated with physical function and the institutionalization of older individuals [60]. The same authors also showed a dramatic interindividual variability in the fecal microbiota of elderly subjects [61].

Aging is thus associated with specific changes in gut microbiota, which have been demonstrated also by other studies reviewed elsewhere [62,63]. Briefly, a lower number of species, decrease in the representation of taxa with purported health-promoting activity, and expansion of *Anaerotruncus, Desulfovibrio, Coprobacillus* and Gram-negative opportunistic pathogens are the most important changes that have been demonstrated in different clinical settings [64,65,66,67]. These distinctive features of older persons’ gut microbiome allow hypothesizing its involvement in the aging process with multiple mechanisms [61], which are summarized in Table 1. In fact, the healthy gut microbiota can modulate immune cell function, metabolic balance, insulin sensitivity, and the host gene expression through multiple mediators, including short-chain fatty acids (SCFA), antioxidants, and pro-inflammatory cytokines [68,69]. Nutrition may play a key role in this process [63,68,69], since most of the mediators synthetized by gut bacteria are derived from dietary intake. Moreover, the differences in gut microbiota that Claesson et al. observed between community dwellers and nursing-home residents were not independent of dietary habits [60,61]. As such, the microbiota could simply be a mediator between nutrition and the ageing phenotype.

Interestingly, studies assessing the fecal microbiota composition of healthy centenarians and supercentenarians have demonstrated that the composition of their core microbiota includes a wide representation of taxa, such as *Faecalibacterium prausnitzii*, *Eggerthella*, *Anaerotruncus*, *Bilophila*, *Akkermansia* and *Butyricimonas*, with beneficial metabolic activities [70,71,72]. These activities include short-chain fatty acid (SCFA) production, which is associated with many of the processes listed in Table 1, including insulin sensitivity, the modulation of inflammation, and the promotion of anabolism [73,74]. Thus, these bacteria have been proposed as microbial biomarkers of healthy, active aging [70].

### 2.4. Gut Microbiota and Physical Frailty

Most of the existing research on the relationship between aging and gut microbiota has been carried out in an epidemiological context, without a specific focus on comprehensive geriatric assessment. Yet, some evidence associating physical frailty with altered gut microbiota composition exists. A summary is provided in Table 2.

First, in their pioneering study on the ELDERMET cohort (www.eldermet.ucc.ie) Claesson et al. demonstrated that the species richness of the fecal microbiota of older subjects is inversely related to physical performance [60]. A secondary analysis of the same cohort has recently revealed that, in community dwellers, the presence of frailty, as measured through the Barthel Index (BI), is associated with a gut microbiome profile similar to that typical of nursing-home residents, with an increased representation of *Anaerotruncus*, *Desulfovibrio*, and *Coprobacillus* [64].

In a group of 728 female twins (mean age 63, range 42–86), Jackson et al. [75] found an inverse correlation between the Rockwood Frailty Index and gut microbiome alpha diversity, which is an expression of the number of bacterial species detected in each fecal sample by metagenomic analyses. They also found that the fecal average relative abundance of 12 bacterial species was significantly correlated with the Rockwood Frailty Index. Namely, *Faecalibacterium prausnitzii* exhibited an inverse correlation, while *Eubacterium dolichum* and *Eggerthella lenta* exhibited a positive correlation. Interestingly, very similar results have been obtained by Maffei et al. in a cohort of 85 community dwellers aged between 43 and 79 [76]. Finally, data from our research group referring to 76 older patients (mean age 83) hospitalized for acute extraintestinal disease showed that the average relative abundance of seven bacterial taxa was significantly associated with the Rockwood Clinical Frailty Scale [66].

These results are not merely speculative; they have important clinical correlates. For example, gut microbiota dysbiosis can be associated with a reduced survival in older individuals with frailty or disability [66]. Moreover, the over-representation of opportunistic pathogens in the gut microbiota of frail multimorbid older patients may also increase the risk of developing infections, such as *Clostridium difficile* enterocolitis [65,77].

However, these studies do not establish any cause–effect relationship between gut microbiota dysbiosis and physical frailty, due to their cross-sectional design. As such, they may represent very important hypothesis generators for future research. For example, the reduced representation of *Faecalibacterium prausnitzii*—a well-known SCFA producer and metabolic modulator—in frail subjects supports the hypothesis of gut microbiota involvement in the onset of physical frailty [78]. It is also noteworthy that animal models of healthy aging and longevity exhibit a wide representation of SCFA producers and species that are able to degrade complex carbohydrates in their gut microbiome, whose composition is similar to that detected in human centenarians [79]. Similarly, recent studies have demonstrated that some microbiome-derived indoles are able to extend health span irrespective of longevity in animal models of aging [80]. All of this evidence supports the assumption that the gut microbiome is involved in aging phenotype and in the onset of physical frailty.

Interestingly, two human studies have reported significant correlations between longitudinal shifts in gut microbiota composition and the decline in cognitive function of middle-aged obese subjects, healthy older individuals, and patients with cirrhosis [81,82]. These findings support the hypothesis that a “gut–brain axis”, already demonstrated for a wide spectrum of psychiatric and neurological diseases [83], is also involved in the onset of cognitive frailty and, possibly, dementia. In older individuals, there are strict physiopathological connections between brain and muscle function, so that it is often impossible to discern physical and cognitive frailty, which influence one another in a sort of “loop” that ultimately leads to disability [84]. In this context, gut microbiota could influence physical performance and muscle function through the mediation of the central nervous system.

## 3. The Rationale for a Possible Correlation between Gut Microbiota and Sarcopenia

### 3.1. The Influence of Exercise on Gut Microbiota: Animal Studies

In current state-of-the-art literature, there is evidence supporting the concept that gut microbiota composition is modulated by physical exercise [85]. Several animal studies have shown that exercise is associated with increased gut microbiota biodiversity and the modulation of systemic and local gut inflammation. Exercise can promote these changes synergistically with diet, so that an unhealthy diet can antagonize the beneficial effects of exercise, and vice versa.

In mouse models treated with high-fat diets to induce obesity, high-intensity training prevented the onset of obesity-associated gut microbiota dysbiosis, keeping the overall biodiversity high [86]. The exercise-induced modifications of gut microbiota in obese rats are also different from that induced by a healthy diet, suggesting a specific signature induced by exercise on the microbial communities, including the expansion of taxa such as *Faecalibacterium*, *Clostridium*, and *Allobaculum* [87,88]. Studies comparing the effects of exercise vs. diet in obese rats have also demonstrated that the effects of exercise on gut microbiota are stronger and more stable over time, promoting more effectively intestinal mucosa integrity and metabolic function [88,89]. High-intensity exercise-induced modifications in gut microbiota are even able to modify the intestinal response to chemically-induced colitis after fecal transplantation in gnotobiotic mice [90]. However, other studies have shown that the exercise-induced beneficial changes in the gut microbiota of mice are partly blunted by the introduction of a high-fat diet [91], supporting the view of an “integrated cross-talk” between exercise and nutrition in shaping gut microbiota composition.

In mouse models, even moderate-intensity exercise can determine significant and reproducible changes in gut microbiota composition, including increased biodiversity and the representation of key taxa with health-promoting metabolic activities, such as *Butyricimonas*, *Prevotella*, and *Akkermansia* [92,93].

### 3.2. The Influence of Exercise on Gut Microbiota: Human Studies

Only a few studies have demonstrated exercise-induced changes on gut microbiota in humans, and most evidence comes from observational studies. Bressa and colleagues recently observed a significant over-representation of some health-promoting bacterial species, including *Akkermansia*, *Faecalibacterium*, and *Roseburia*, in fecal samples of adult women with active lifestyles compared with sedentary age-matched women [94]. These results confirmed the findings by Clarke et al., who compared the fecal gut microbiome composition of professional rugby athletes with sedentary groups matched for body size, age, and gender [49]. Rugby athletes in fact had a significantly higher microbiome biodiversity, and increased representation of 22 distinct taxa, including *Akkermansia*, than controls. The representation of these taxa in stool samples was also correlated with dietary protein intake [49], confirming once again the strict liaison between nutrients and exercise in shaping gut microbiota composition.

More recently, Barton et al. showed that the differences in gut microbiome composition between professional rugby players and controls are also matched by significant differences in the fecal metabolome. In fact, athletes showed a higher representation of SCFA-producing bacteria and bacterial genes involved in carbohydrate and amino acid metabolism than controls, resulting in higher fecal concentrations of acetate, butyrate, and propionate [95]. The key roles of *Prevotella*, *Akkermansia*, and SCFA producers have also been confirmed by analyses carried out on the fecal microbiota of competitive cyclists [96].

Additionally, the gut microbiota composition correlates with measures of cardiorespiratory fitness in both premenopausal women [97] and healthy young volunteers [98]. Adequate exercise training is also associated with partial recovery of the gut dysbiosis associated with the myalgic encephalitis/chronic fatigue syndrome [99].

All of these studies were carried out on adult subjects, lacking a specific focus on older individuals. However, given the high prevalence of sarcopenia in this age range and the effectiveness of exercise as a countermeasure to prevent or treat sarcopenia, more studies are needed in order to clarify whether microbiota can be an active mediator of the benefits of physical exercise in older age. However, some evidence from animal models suggests that the exercise-induced modifications in gut microbiota could be mitigated in aged individuals [100].

### 3.3. Gut Microbiota and Systemic Effects Involving Muscle Function

Gut microbiota has been recently defined as a “transducer” of nutrient signals for the host [101]. In fact, it is strongly influenced by diet, and is able to produce mediators that influence metabolic balance, insulin sensitivity, and inflammation.

These concepts move from the observation by Backhed et al. that germ-free mice persistently exhibit a lean phenotype even when fed high-calorie, high-fat diets [102]. In fact, gut microbiota is able to give pro-anabolic signals to the host by producing diet-derived mediators. In germ-free mice, gut microbiota transplantation is able to promote anabolic activation in the bone, with improved bone mineral density [103]. The involved microbial metabolites are SCFA, promoting systemic IGF-1 release after mucosa absorption [103]. IGF-1 is a well-known anabolic hormone with important actions also regarding the modulation of the inflammatory response [104], and may represent a key element in the systemic effects of gut microbiota.

Moreover, the transplantation of the fecal microbiota from African malnourished children to mice resulted in mouse failure to thrive, while transplantation of the fecal microbiota from well-nourished children to mice did not alter growth [105]. The results of these experiments confirm that a healthy gut microbiota promotes anabolism, while dysbiotic microbiota is associated with anabolic resistance or even catabolism [101].

These findings are not surprising when considering how many nutrients are made bioavailable for the host by microbiota metabolism, or are significantly produced by the microbiota itself (Table 3) [101]. Interestingly, the studies linking Mediterranean-style dietary patterns with gut microbiota composition in adult community dwellers have demonstrated that many microbial mediators derived from nutrients can be found in urine, blood, and feces [52,53,54,55]. This circumstance confirms the active role of the gut microbiota as a metabolic modulator for the host.

### 3.4. Overview of Mediators by Gut Microbiota and Their Effect on Skeletal Muscle

Several compounds produced or modified by the gut microbiota can enter systemic circulation and ultimately influence skeletal muscle cells (Table 3). For example, a healthy gut microbiota is able to produce significant amounts of folate and vitamin B_12_ [106], which may improve muscle anabolism and prevent hyperhomocysteinemia-induced oxidative stress and endothelial damage, leading to reduced muscle function [107]. Moreover, the gut microbiota is able to synthesize some amino acids, such as tryptophan, representing the fundamental substrates for muscle protein anabolism [108]. Tryptophan may also stimulate the IGF-1/p70s6k/mTor pathway in muscle cells, promoting the expression of genes involved in myofibrillar synthesis [109]. Betaine, a microbial metabolite derived by glycine betaine provided by diet, can activate cytosolic calcium influx, Extracellular-signal–Regulated Kinase (ERK) signaling, and IGF-1 synthesis in human osteoblast cultures, which enables hypothesizing its effect on skeletal muscle cells [110]. More generally speaking, the effect of several nutraceuticals with documented promotion of anabolism in skeletal muscle cells may be mediated by gut microbiota metabolism [111].

The most studied putative mediators of the effect of gut microbiota on skeletal muscle function are SCFA [112]. These substances are generally derived from the bacterial metabolism of nutrients, such as proteins, which are introduced with diet [108]. Their main host targets are skeletal muscle mitochondria [113]. In fact, the SCFA produced by gut bacteria such as *Faecalibacterium*, *Succinivibrio*, and *Butyricimonas* can enter systemic circulation and be absorbed by skeletal muscle cells, where they act as ligands for free fatty acids receptors 2 and 3 (FFAR-2 and FFAR3) [73,74]. These receptors have a key role in modulating glucose uptake and metabolism, and in promoting insulin sensitivity [113]. Moreover, SCFA upregulate the NAD-dependent deacetylase sirtuin-1 (SIRT1) receptor, which is a modulator of mitochondrial biogenesis [114,115]. Interestingly, the expression of mitochondrial proteins is positively correlated with the average relative abundance of SCFA producers in the gut of subjects with inflammatory bowel disease, providing an evidence of a strict connection between microbiota and mitochondrial function [116]. The involved mitochondrial proteins may determine the efficiency of energy production, redox balance, and the modulation of the inflammatory cascade activation [112]. A low representation of SCFA producers in gut microbiota has been associated with increased subclinical chronic inflammation, which reinforces the anabolic resistance [62].

Among SCFA, the most interesting mediator from a skeletal muscle perspective is butyrate. Besides its known anti-inflammatory properties, which are demonstrated in inflammatory bowel diseases [117], this metabolite may be responsible for the activation of several regulatory pathways (for example, UCP2-AMPK-ACC and PGC1-α), resulting in increased ATP production, and ultimately, in the improved metabolic efficiency of myofibers [118]. Butyrate also acts through the inhibition of histone deacetylase, which leads to apoptosis prevention and protection against muscle protein catabolism [119]. Walsh et al. have in fact demonstrated that the administration of butyrate to aged mice is associated with the prevention of physiological age-related muscle mass loss [120].

Conversely, there are data that speak against the beneficial metabolic activity of acetate, which is another SCFA derived from gut microbiota metabolism. Its systemic concentrations have been correlated with increased insulin resistance and obesity [120,121], whose presence is detrimental for skeletal muscle anabolism, and may have negative consequences on sarcopenia.

Skeletal muscle cell mitochondrial biogenesis may theoretically be regulated also by secondary biliary acids, which are synthesized by gut microbiota from primary bile acids [112]. However, at present, there is no direct proof of anabolic modulation of biliary acids on skeletal muscle cells.

A paradigmatic example of how nutrients can be modified by the gut microbiota to produce substances that may have an important influence on muscle function is represented by ellagitannins. Ellagitannins are a class of polyphenols that are abundant in fruits and nuts such as pomegranates, black raspberries, raspberries, strawberries, and walnuts. They are very poorly absorbed at the small intestinal level and, upon ingestion, reach the large intestine, where they are extensively metabolized by the gut microbiota to form smaller phenolic structures known as urolithins [122]. One of the most relevant among these metabolites is urolithin A (Uro-A), which has recently been described as preventing the accumulation of age-related dysfunctional mitochondria and extending the lifespan of *C. elegans* [123]. In the same studies, the investigators demonstrated an improved exercise capacity in two different mouse models of age-related decline of muscle function, as well as in young rats, after consumption of Uro-A. The authors have also successfully completed a clinical phase 1 trial demonstrating the ability of orally consumed Uro-A to modulate muscle and mitochondrial biomarkers in a randomized placebo-controlled durable blind framework [124,125]. This case represents an amazing example of a metabolite generated by the interaction of dietary phytochemicals and the human gut microbiota, which has demonstrated activity towards both genes and metabolites linked to muscle function in humans.

### 3.5. Muscular Effects of Gut Microbiota Manipulation: Animal Studies

Few studies have evaluated the effects of gut microbiota modifications on parameters of muscle mass and function, and most were carried out on animal models. Varian et al. have demonstrated that the administration of a probiotic containing *Lactobacillus reuteri*—a known modulator of transcriptional factor Forkhead Box N1 (FoxN1)—to mouse models of cancer is able to inhibit the development of cachexia, and is associated with the preservation of muscle mass [126].

Some probiotics also have a marked anti-inflammatory effect, which may have beneficial consequences for muscle health through the promotion of anabolism. For example, treatment with probiotic formulations containing *Faecalibacterium prausnitzii*, one of the main SCFA producers, was associated with improved liver anabolism and reduced systemic inflammation in mice [127]. Similarly, aged mice treated with spirulina, a cyanobacterium used as a food additive or supplement, experienced improvement in systemic biomarkers of inflammation and oxidative stress [128].

Finally, some studies on animal models have also evaluated the systemic effects of gut microbiota homeostasis disruption induced by antibiotic administration. Guss et al. recently demonstrated that gut microbiota dysbiosis is associated with impaired bone strength and mechanical properties, possibly resulting from a reduction of osteogenesis due to lacking anabolic stimuli [129]. Similarly, Caputi et al. showed that antibiotic-induced dysbiosis promotes the distortion of neuromuscular transmission in mice, which in turn may promote muscle protein catabolism [130].

### 3.6. Muscular Effects of Gut Microbiota Manipulation: Human Studies

The only intervention study carried out on older patients and targeted at exploring the effects of gut microbiota modifications on skeletal muscle outcomes involved the administration of prebiotics, i.e., substances promoting the overexpression of beneficial bacteria. In their randomized controlled trial, Buigues et al. enrolled 60 older patients who received treatment with a prebiotic formulation including fructooligosaccharides and inulin versus placebo for 13 weeks. Surprisingly, the treatment group experienced improvement in two outcomes of muscle function: exhaustion and handgrip strength [131]. Thus, these data support the hypothesis of a modulation of muscle function by gut microbiota. Unfortunately, no other studies have explored this field to date.

## 4. Conclusions: A Gut–Muscle Axis in Aging?

**Figure 1:**
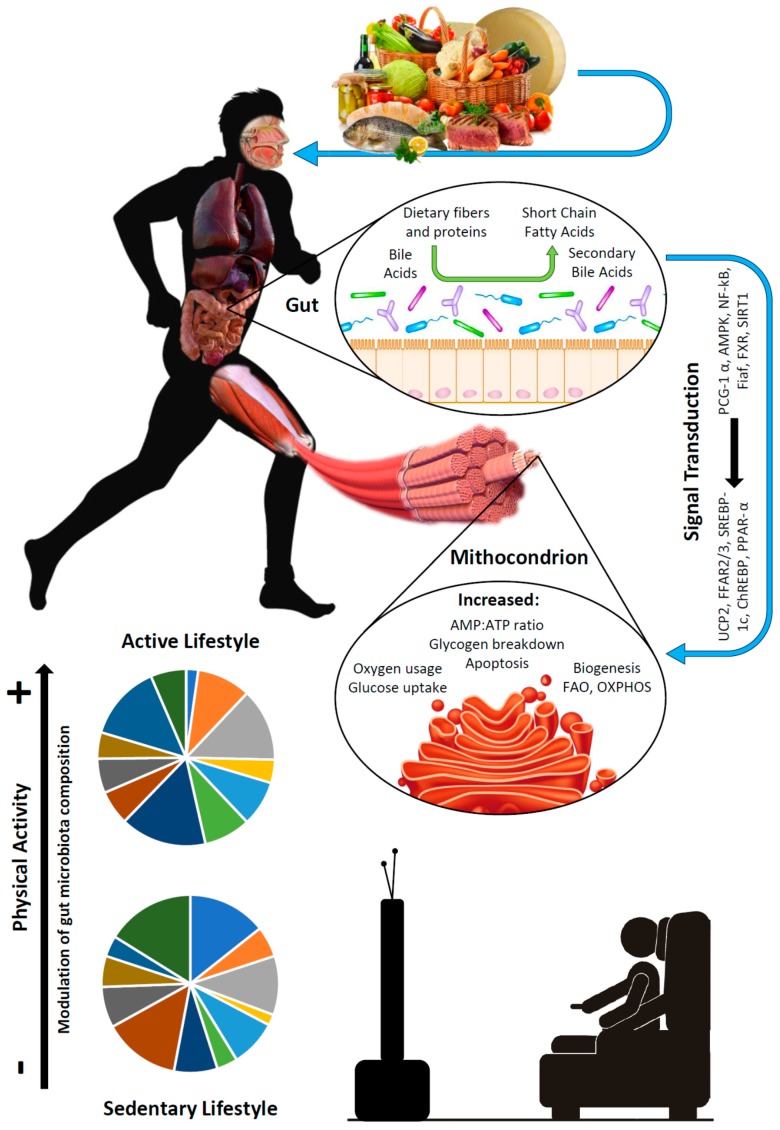
Overview of the putative physiopathological mechanisms that put gut microbiota composition at the cross-road between nutrition and muscle function. Diet influences the microbiota composition; in turn, microbiota metabolizes some nutrients, including fibers and proteins, into mediators, such as short-chain fatty acids, which enter the systemic circulation. These mediators have a known influence on myocytes, and namely on their mitochondria, through multiple signaling pathways that result from the modulation of inflammation and the promotion of insulin sensitivity. The lower part of the figure also shows that physical exercise itself can modulate gut microbiota composition and represent a relevant player in these phenomena.

The current state-of-the-art literature supports the hypothesis that gut microbiota may be involved in the onset and clinical course of sarcopenia [85,112]. Since nutrition is one of the main determinants of gut microbiota composition, and is also involved in the pathogenesis of sarcopenia, the gut microbiota may be at the physiopathological cross-road between these two elements (Figure 1). Some key microbial taxa may have a relevant role in determining muscle structure and function by producing metabolic mediators that influence the host physiology after intestinal mucosa absorption. Glycine betaine, tryptophan, biliary acids, and SCFA, namely butyrate, are the most promising of these putative mediators. These concepts are summarized in Figure 1. As such, these molecules—and the bacteria that produce them in the fecal microbiota—could theoretically represent promising biomarkers of sarcopenia, whose detection is recognized worldwide as a key priority in the field of sarcopenia research [132].

However, studies specifically assessing gut microbiota composition in physical frailty and sarcopenia are lacking. Intervention studies that test the skeletal muscle effects of prebiotic or probiotic administration have been focused mainly on animal models, and translation of their results in humans is uncertain. Moreover, the currently available commercial probiotic preparations are in many cases not targeted at the gut microbiota alterations that can be detected by metagenomics, although the development of novel “next-generation” probiotics seems very promising in this field [133].

In this scenario, future studies should be developed to test the presence, functionality, and clinical relevance of the putative gut–muscle axis, correlating gut microbiota composition with nutrition, muscle performance, and structure, which nowadays can be easily assessed using low-cost tools [134,135]. The effects of dietary manipulations, including the beneficial effects of a Mediterranean-style diet and prebiotic and probiotic administration, should be also assessed in older individuals while considering skeletal muscle functionality and clinical variables as outcomes. In other terms, a clinical transition of the basic science background linking gut microbiota with muscle physiology should be one of the priorities of aging nutrition, frailty, and sarcopenia research.

## Figures and Tables

**Table 1:** Overview of the main effects of a healthy gut microbiota on the physiologic processes involved in healthy, active aging.

Effect	Mediators/Mechanisms	Target Cells/Systems
Suppression of chronic inflammation, modulation of inflamm-aging	Down-regulation of Interleukin-6, Interleukin-8, Interleukin-10, Tumor Necrosis Factor-α	Neutrophils, activated lymphocytes, natural killer cells
Enhancement of antioxidant activity	Diet-derived polyphenols, ellagitannins, B complex vitamins	All of the host’s cells
Prevention of insulin resistance	Short-chain fatty acids, conjugated linoleic acid, gut peptides	Adipocytes, myocytes
Maintenance of gut barrier function	Reduced absorption of lipopolysaccharide and pro-inflammatory bacterial endotoxins	Neutrophils, activated lymphocytes, natural killer cells
Enhancement of xenobiotic metabolism and detoxification	Reduced absorption of xenobiotics by increased degradation in the gut	All of the host’s cells
Modulation of host gene expression	Butyrate, other bacterial metabolic products	Skeletal muscle, central nervous system, immune cells

**Table 2:** Summary of studies exploring the association of fecal microbiota composition with frailty or disability in older individuals.

First Author, Journal, Year [Ref.]	Country	Study Design	Sample Size	Setting/Health Status	Method of Measuring Frailty or Disability	Mean Age (Years)	Main Findings
Claesson MJ, Nature, 2012 [52]	Ireland	Cross-sectional	178	83 community dwelling; 20 outpatient clinic; 15 short-term rehabilitation; 60 nursing homes	Barthel Index	78	Decreased species richness with increased functional dependence measured by the Barthel Index Nursing home residents had a recognizable microbiome cluster Increased abundance of *Bacteroides* and decreased abundance of *Oscillibacter*, *Ruminococcus*, and *Prevotella* with increasing frailty
Jeffery IB, ISME J, 2016 [56]	Ireland	Prospective	384	Community; outpatient clinic; short-term rehabilitation; nursing homes	Barthel Index	78	The presence of frailty (measured by the Barthel Index) was correlated with reduced species richness and a composition of gut microbiota that is similar to that detected in nursing home residents
Jackson MA, Genome Med, 2016 [67]	United Kingdom	Cross-sectional	728	Community-dwelling twins	Rockwood Frailty Index	63	Frailty was negatively associated with gut microbiota biodiversity The relative abundance of 22 taxa of gut microbiota was associated with frailty (negative association: *Faecalibacterium prausnitzii*; positive association: *Eubacterium dolichum*, *Eggerthella lenta*)
Maffei VJ, J Gerontol A Biol Sci Med Sci, 2017 [68]	United States	Cross-sectional	85	Community-dwelling volunteers	Frailty Index questionnaire validated by authors	63	Inverse correlation between frailty index and gut microbiome biodiversity Frailty is associated with an increased interindividual variability of gut microbiota Frailty is associated with an increased abundance of *Coprobacillus* and *Dialister*, and a reduced abundance of Rikenellaceae, *Paraprevotella*, and *Sutterella* Frailty is associated with a lower bacterial metabolic activity
Ticinesi A, Sci Rep 2017 [58]	Italy	Cross-sectional	76	Inpatients hospitalized for extraintestinal acute disease	Rockwood Clinical Frailty Scale	83	Frailty is not associated with gut microbiome biodiversity Frailty is associated with the increased representation of seven gut microbiome taxa, including *Fonticella*, *Oscillospira*, *Peptococcus*, *Porphyromonas*, and *Prevotella*

**Table 3:** Overview of the main microbial metabolites acting as nutrients or metabolic/physiological modulators for the host, which are also possibly involved in skeletal muscle function.

Substance	Bacterial Taxa Involved	Possible Effects on Muscle
Folate	*Bifidobacteria* *lactobacilli*	Biosynthesis of amino acids DNA synthesis, methylation, and repair
Riboflavin (vitamin B_2_)	*Bacillus subtilis* *Escherichia coli* *Bifidobacteria*	Improvement of redox reactions and energy production Improved resistance to fatigue
Vitamin B_12_	*Propionibacteria* *Lactobacillus reuteri*	Preservation of strength through the prevention of homocysteine-induced oxidative stress and endothelial damage
Glycine betaine	*Escherichia coli* *Klebsiella*	Stimulation of anabolism and cell proliferation by IGF-1 synthesis
Tryptophan	Several bacterial species	Stimulation of anabolism and cell proliferation by IGF-1 synthesis
Short-chain fatty acids	*Faecalibacterium* *Butyricimonas* *Succinivibrio* *Pseudosuccinivibrio*	Promotion of insulin sensitivity, modulation of inflammation, promotion of mitochondrial biogenesis, and energy production
Urolithins	Several bacterial species involved (not fully identified)	Preservation of skeletal muscle cell mitochondrial biogenesis and activity, promotion of muscle anabolism

IGF-1: insulin-like growth factor-1.
